# A Tiny Protein Plays a Big Role in DNA Repair

**DOI:** 10.1371/journal.pbio.0040184

**Published:** 2006-05-09

**Authors:** Liza Gross

The human genome lies deep within the cell interior, sequestered behind the double-walled membrane of the nucleus, yet still remains vulnerable to a wide variety of hazardous materials. Two of DNA's worst enemies, ultraviolet light and chemical carcinogens, can wreak havoc on the molecule by mutating individual nucleotides or changing its physical structure. In most cases, genomic integrity is restored by specialized suites of proteins dedicated to repairing specific types of injuries.

One mending mechanism, called nucleotide excision repair (NER), recruits and coordinates the services of roughly 25 proteins to recognize and remove structure-impairing lesions, including those induced by ultraviolet light. At the center of this effort is the ten-subunit transcription/repair factor IIH (TFIIH) complex. As its name suggests, in addition to its role in DNA repair, it also regulates transcription; how TFIIH coordinates these very different activities is still under investigation. Regardless, three TFIIH genes— *XPB, XPD*, and *TTDA*—have been implicated in the photosensitive form of a rare inherited premature aging syndrome called trichothiodystrophy (TTD), which is characterized by brittle hair and nails, scaly skin, and neurological degeneration.

In a new study, Giuseppina Giglia-Mari, Jan Hoeijmakers, Catherine Miquel, Wim Vermeulen, and colleagues created a fluorescently tagged version of trichothiodystrophy group A (TTDA) to investigate its role in repair and transcription. By experimentally modifying the transcription function and by triggering DNA repair in human cell lines expressing the fluorescent TTDA protein, the researchers show that TTDA first of all dynamically associates with TFIIH, and that TTDAs become stably incorporated only while the complex is engaged in NER.

NER steps in when damage interferes with the elongation of a newly transcribed gene, promoting cell survival. It also plays an anti-cancer role by surveying the genome for damage. In ultraviolet-induced NER, TFIIH unzips the double helix to access the injury, and then recruits four proteins to stabilize the open strand, evaluate the damage, and cleave the DNA on either side of the lesion. Other proteins produce new nucleotides to fill the gap. Cells collected from patients with TTD-A have *TTDA* mutations that produce either truncated, nonfunctional versions of the protein or no protein at all, resulting in reduced TFIIH levels. TTD-A cells also have NER defects and are hypersensitive to ultraviolet light.

To study the role of the TTDA subunit in the TFIIH complex's functions, the researchers tagged TTDA and the XPD subunit with green fluorescent protein (GFP) to monitor and compare their movement and behavior using high-resolution confocal microscopy. Both TTDA-GFP and XPD-GFP could stably incorporate into TFIIH and function in DNA repair, proving reliable tools for studying TFIIH's spatial and temporal distribution and activity during transcription and DNA repair.

Both TTDA-GFP and XPD-GFP were observed in the cytoplasm and nucleus, in contrast to the XPB subunit, which is known to localize only in the nucleus. To determine if TTDA and XPD assemble with TFIIH in the cytoplasm, the researchers used a customized version of a motility-monitoring technique called fluorescence recovery after photobleaching (FRAP). Applying a high-intensity laser to a specific region in a cell destroys fluorescence in that area; as unaffected GFP-tagged molecules from other regions replace the zapped molecules, fluorescence is recovered. Recovery is much faster for free-moving GFP-tagged proteins than for those caught up in interactions with other cellular proteins.

In the cytoplasm, TTDA-GFP moved much faster than XPD-GFP, but did not interact with each other. Both proteins, however, were less motile in the nucleus, where they interacted with TFIIH. These results indicated that TTDA exists in two pools in non-irradiated cells, one free and one bound to TFIIH. After irradiation, however, fluorescence was not completely recovered for either TTDA-GFP or XPD-GFP, suggesting a lack of motility one would expect by participating in DNA repair. When the researchers inhibited a protein essential for NER initiation in the irradiated cells, they observed a reduced fraction of immobilized TTDA-GFP, confirming that TFIIH must incorporate TTDA-GFP (thus, reducing its mobility) for NER. By interfering with the transcription machinery, they go on to show that TFIIH does not stably incorporate TTDA when TFIIH is simply bound to DNA; TFIIH only enhances association with TTDA during NER.

Altogether, these results show that NER-specific lesions induce TTDA to form a stable association with the TFIIH in living cells. Giglia-Mari et al. propose that once TTDA nestles into place after the core TFIIH complex attaches to a lesion, it triggers a conformational change that recruits the other subunits required for repair. TTDA may also help TFIIH fold properly, preventing it from being degraded and allowing it to accumulate to the levels necessary for NER function. TFIIH molecules spend far longer at NER sites (four to five minutes) than they do at transcription sites (two to ten seconds), suggesting why TFIIH concentrations are so important to NER—and why people that can't produce TTDA experience such debilitating symptoms.

## 

**Figure:**
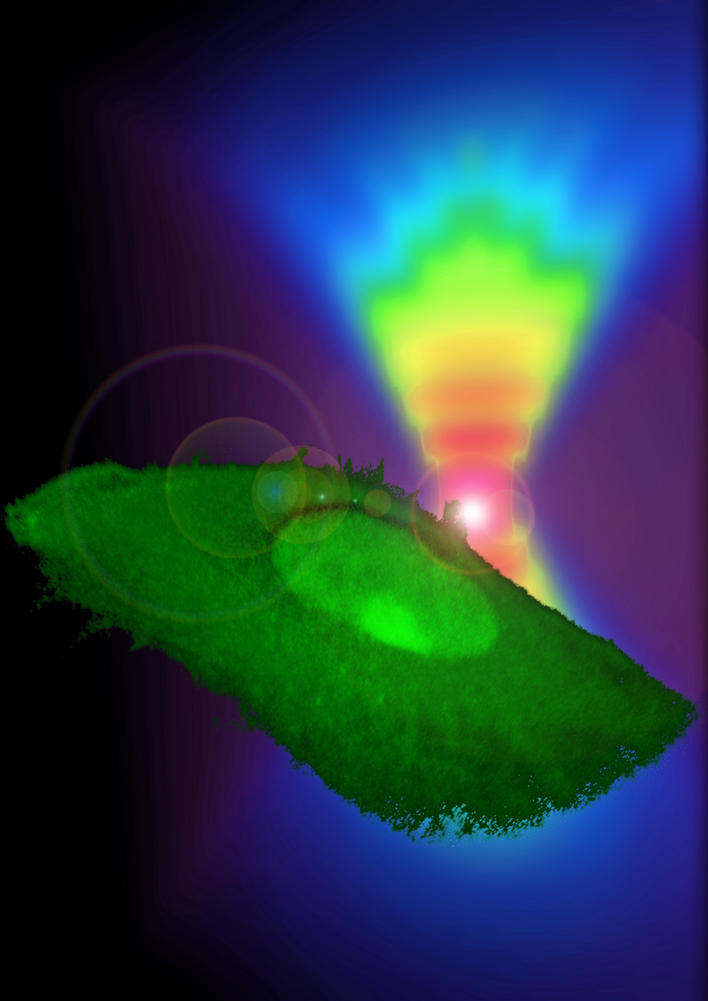
Fluorescence accumulation in the nucleus indicates TTDA retention during the repair reaction at a locally ultraviolet-irradiated area in a living human fibroblast in this three-dimensional reconstruction. (Image: Pierre Olivier Mari and Giuseppina Giglia-Mari)

